# Treatment of Idiopathic Inflammatory Orbital Syndrome (IOIS) With Prominent Lacrimal Gland Involvement Using Oral Non-Steroidal Anti-Inflammatory Drugs (NSAIDs)

**DOI:** 10.7759/cureus.67981

**Published:** 2024-08-27

**Authors:** Hwee Minn Ling, Kit May Chow

**Affiliations:** 1 Department of Ophthalmology, Sultan Haji Ahmad Shah Hospital, Kuantan, MYS

**Keywords:** non-steroidal anti-inflammatory drugs (nsaids), idiopathic orbital inflammatory disease, lacrimal gland enlargement, acute dacryoadenitis, steroid use, oral steroid

## Abstract

Idiopathic orbital inflammatory syndrome (IOIS) is a chronic inflammatory process of unknown etiology, which can either be localized or diffuse. In cases where there is isolated inflammation of the lacrimal gland, it is known as dacryoadenitis. This study focuses on the treatment of a patient with IOIS with prominent lacrimal gland involvement. The mainstay of treatment for idiopathic isolated dacryoadenitis is oral corticosteroids, but non-steroidal anti-inflammatory drug (NSAIDs) is known to be effective in treating idiopathic dacryoadenitis as well. There is no formal study yet to evaluate the use of NSAIDs in treating idiopathic dacryoadenitis. Here, we report a case of idiopathic isolated dacryoadenitis which was successfully treated with NSAIDs.

## Introduction

Idiopathic orbital inflammatory syndrome (IOIS) is a benign non-infectious, inflammatory process of the orbital region with unknown etiology [1]. The pattern of inflammation can either be localized or diffuse. In localized cases, inflammation can either affect the lacrimal gland (dacryoadenitis), extraocular muscles (myositis), sclera (scleritis), or uvea (uveitis) [1]. It can also diffusely involve the orbital fatty tissues in cases of diffuse inflammation. Idiopathic isolated inflammation of the lacrimal gland is known as idiopathic dacryoadenitis. It is a diagnosis of exclusion. Infectious causes - either viral (e.g., mumps, Epstein-Barr virus, cytomegalovirus) or bacterial infection - and autoimmune disorders need to be ruled out before diagnosing as idiopathic dacryoadenitis [1,2]. Symptoms of dacryoadenitis include swelling of the lateral aspect of the eyelid leading to the characteristic S-shaped ptosis, reduced or increased lacrimal secretion, eye discharge, and occasionally proptosis [1,3]. Other presenting symptoms include tenderness over the superolateral region and injection over the palpebral portion of the lacrimal gland and adjacent conjunctiva on upper eyelid eversion [1]. Systemic corticosteroids are generally considered the mainstay of treatment for idiopathic dacryoadenitis [1]. However, it should only be prescribed after confirming the diagnosis, as it may mask other pathology, such as infection. In mild disease, NSAIDs alone are often effective and can be given prior to initiation of steroid therapy [1,3].

This article was previously presented as a poster at the Pahang Research Day, Malaysia, on October 12, 2023.

## Case presentation

**Figure 1:**
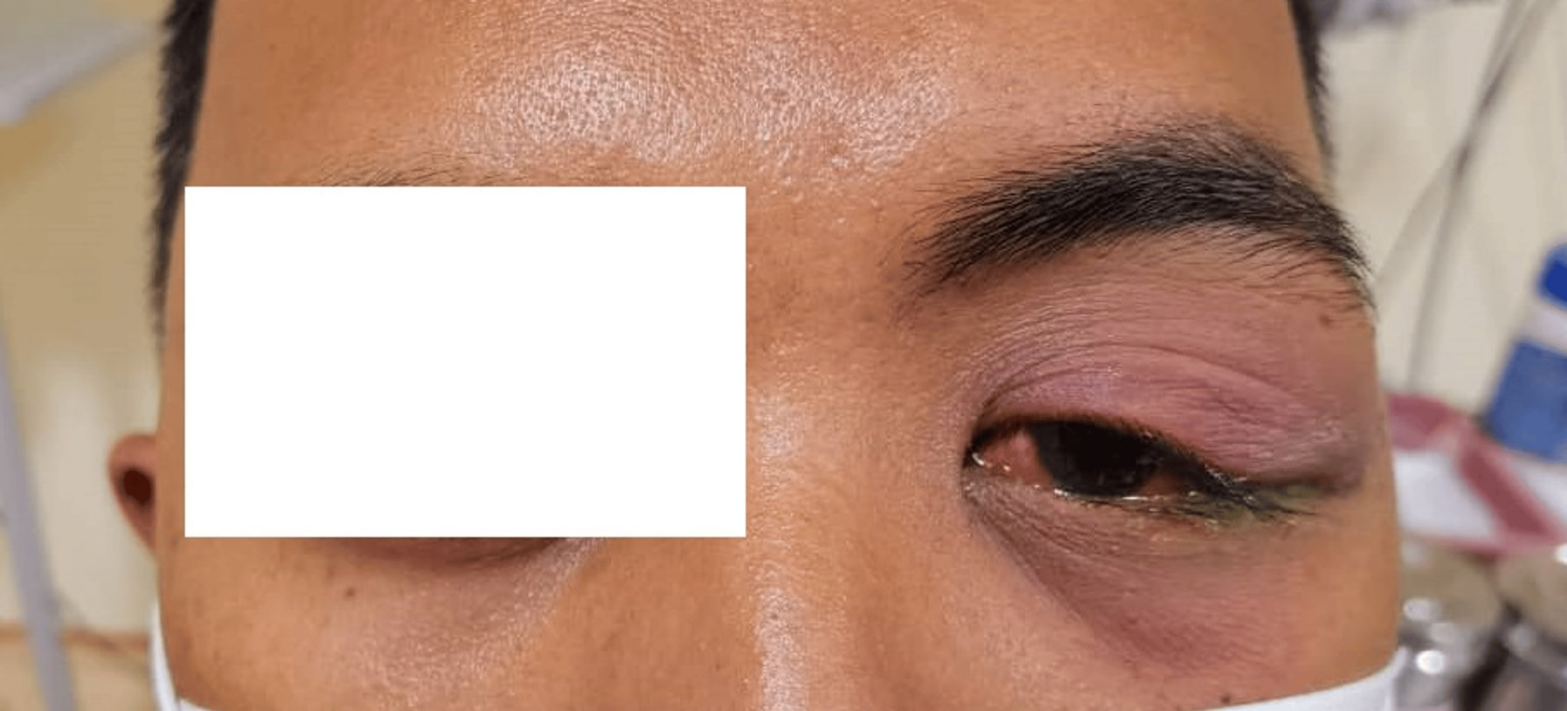
S-shaped ptosis on the first presentation.

A 33-year-old male with no known medical illness, presented with a 10-day history of left superolateral orbital swelling and left eye redness. He was treated in a local government clinic with ointment chloramphenicol and Fucithalmic acid; however, his symptoms did not improve; therefore, he was referred to our center for further assessment and management. Upon taking further history, he denied a history of recent infection, no fever, no eye pain at rest or upon eye movement, no decreased vision, and no double vision. On examination, there was an S-shaped deformity of the left upper lid with ptosis (Figure 1). There was also a boggy swelling over the left lateral orbital ridge with mild tenderness and was warm on palpation. The overlying skin appeared erythematous. Conjunctiva was injected; however, no chemosis was present. There was no restriction in extraocular muscle movement. Hertel exophthalmometer test showed that there was no proptosis. The optic nerve function test showed normal visual acuity, normal color vision, and no relative afferent pupillary defect.

Blood investigations included a complete blood count, which showed a normal total white cell count (6.8 × 10^9^/L), hemoglobin (15.5 g/dL), and platelet count (305 × 10^9^/L); normal C-reactive protein (1.2 mg/L); and a normal erythrocyte sedimentation rate (9 mm/h).

**Figure 2:**
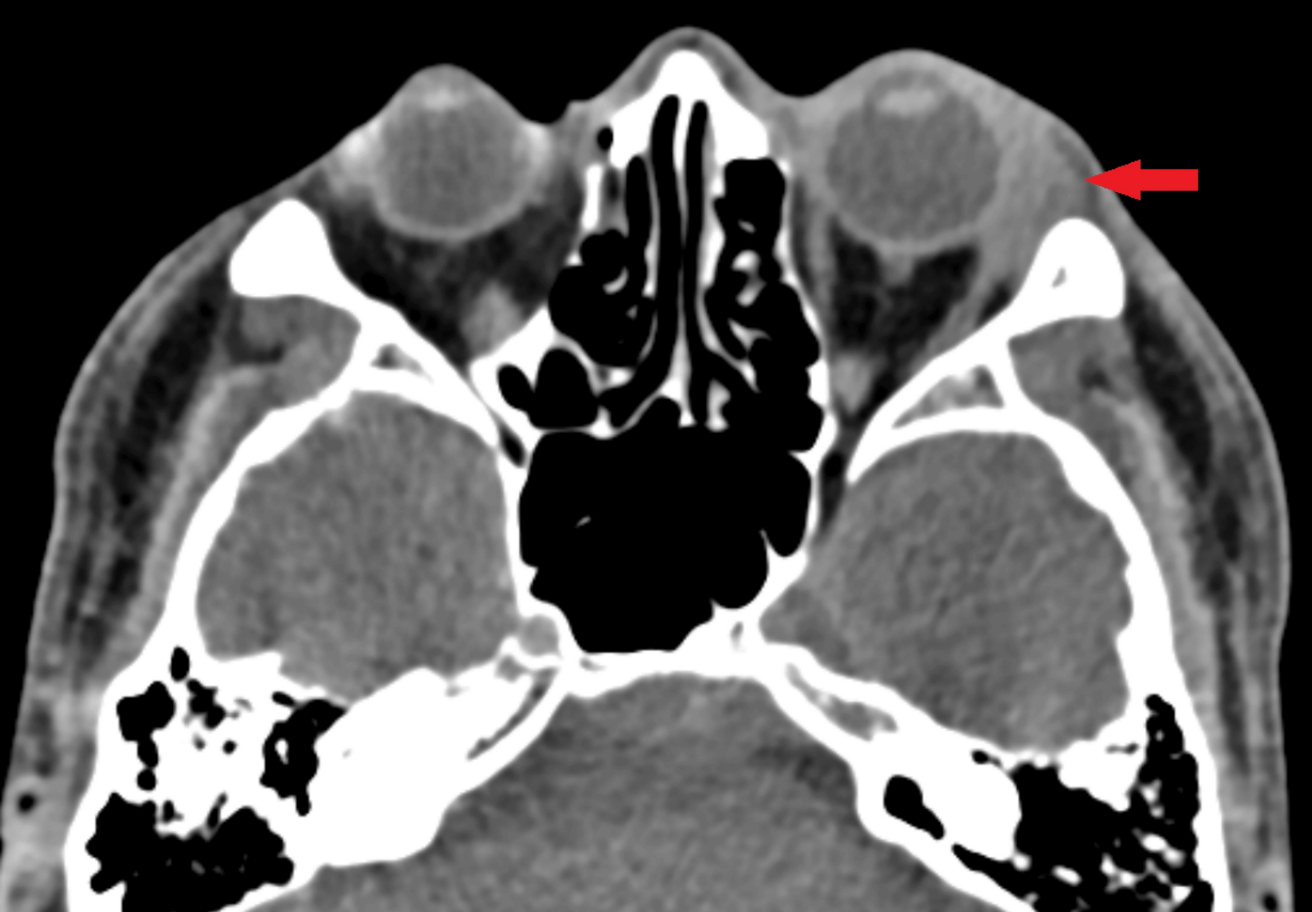
CT orbit showing enlarged left lacrimal gland (arrow).

**Figure 3:**
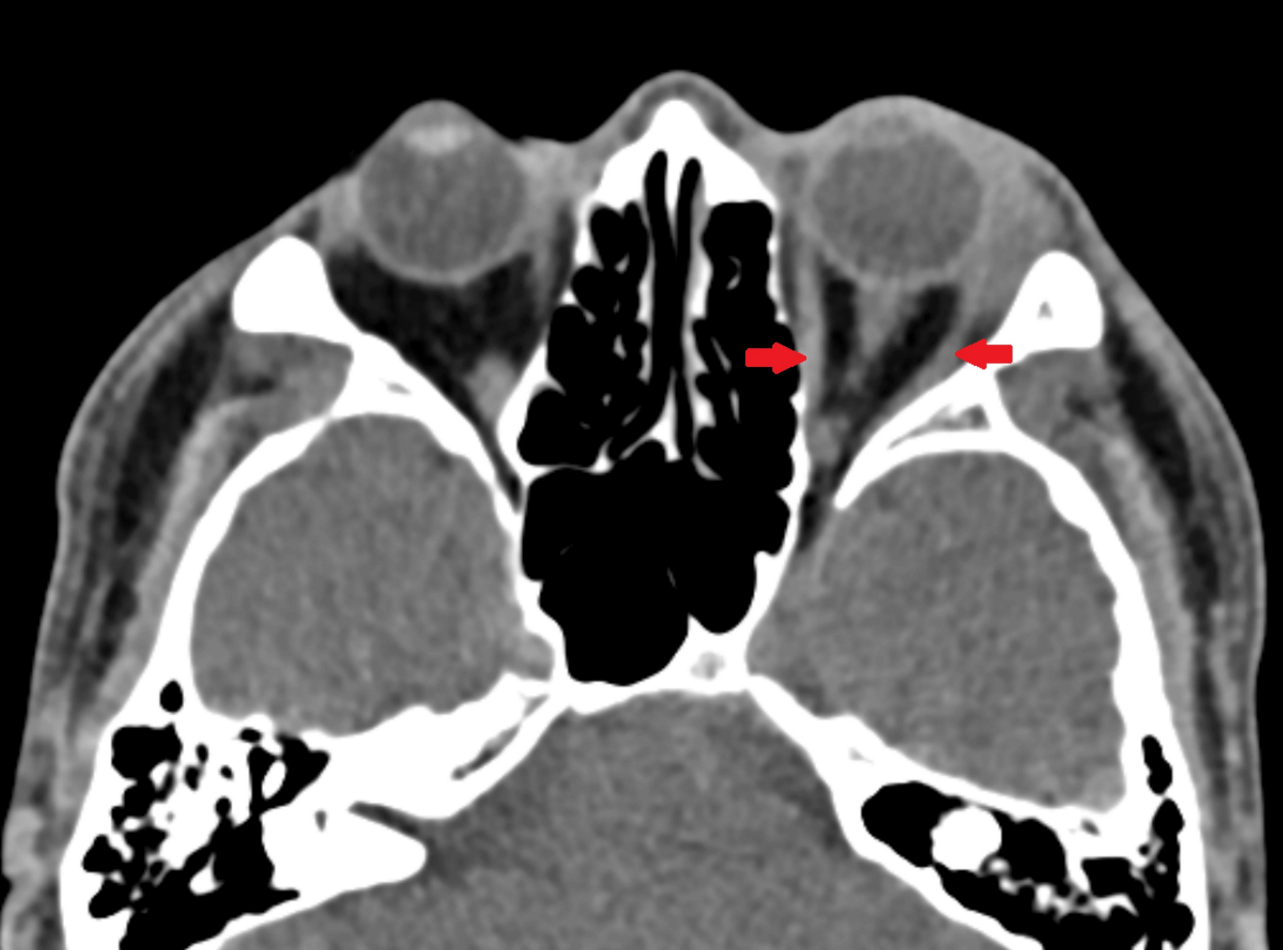
CT orbit showing no tendonitis or myositis (arrows).

**Figure 4:**
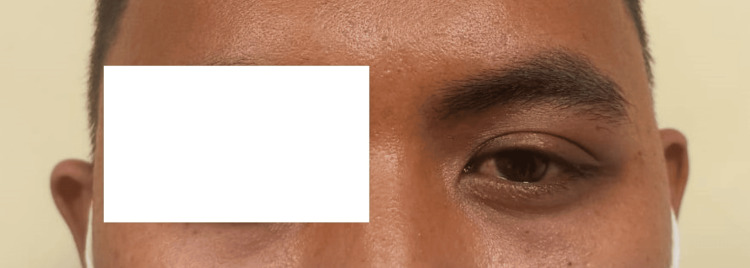
Two weeks after oral ibuprofen.

He was started on Maxitrol (dexamethasone, neomycin sulfate, polymyxin B sulfate) eyedrop, ciprofloxacin eyedrop, and oral amoxicillin/clavulanic acid; however, his symptoms did not improve after one week. We then proceeded with a CT scan orbit which showed an enlarged left lacrimal gland with heterogenous enhancement without tendonitis, myositis, or thickening of uveoscleral (Figures 2, 3). The clinical and radiological findings suggested the diagnosis of left idiopathic inflammatory orbital syndrome (IOIS) with prominent lacrimal gland involvement. Subsequently, he was started on oral ibuprofen. His symptoms resolved two weeks after initiation of oral ibuprofen (Figure 4). 

## Discussion

Some of the differential diagnoses for IOIS are orbital cellulitis, thyroid eye disease, lymphoproliferative disease, and granulomatosis with polyangiitis (GPA) [3]. Without the presence of eye pain, fever, drop in vision, restricted ocular motility, and a full blood count which showed no leukocytosis, it helped to rule out orbital cellulitis [3]. As there were no systemic symptoms like shortness of breath, chronic sinusitis, epistaxis, myalgia, or numbness of fingers and toes, GPA can be excluded [3]. The patient did not have hyperthyroidism symptoms, such as heat intolerance, increased sweating, diarrhea, weight loss, hand tremor, or fatigue. With the help of CT orbit which showed lacrimal gland enlargement without enlarged muscles and tendons insertion or thickening of uveoscleral, we came to the diagnosis of IOID with prominent lacrimal gland involvement or idiopathic isolated dacryoadenitis. However, an important differential diagnosis that should not be missed is lymphoma [3]. It is difficult to differentiate between the two in radiologic imaging. In patients with dacryoadenitis who respond to treatment but dacryoadenitis fails to resolve within three months or if there is a recurrence, a biopsy is necessary [4]. 

Systemic corticosteroids are often considered the gold standard of treatment for idiopathic dacryoadenitis due to their anti-inflammatory and immunosuppressive effects. Most patients will show rapid improvement in their symptoms after initiation of oral corticosteroids. However, systemic corticosteroids can cause a wide array of adverse effects ranging from mild to severe, of which some are unavoidable. Corticosteroids put an individual at higher risk of infections, and these can be either mild or serious life-threatening infections [5]. Other side effects include the risk of adverse gastrointestinal effects, such as gastritis, gastric ulcer formation, and gastrointestinal bleeding, as well as metabolic adverse effects causing an increase in fasting glucose level [5]. Although NSAIDs have been well known for their gastric adverse effects, the risk can be reduced by suppressing acid production using a proton pump inhibitor, such as omeprazole or pantoprazole. In this study, the patient was started on ibuprofen and showed significant improvement after two weeks. As such, the use of systemic corticosteroids as treatment for idiopathic dacryoadenitis can be avoided in this patient.

## Conclusions

Most cases of isolated idiopathic dacryoadenitis respond well to oral corticosteroids. In mild cases, the use of NSAIDs alone can be used as the initial treatment before starting the patient on systemic corticosteroids. In the present case, a patient with idiopathic dacryoadenitis was commenced on NSAID (ibuprofen) instead of systemic corticosteroids. As the patient responded well to oral ibuprofen, usage of systemic corticosteroids, which is often associated with multiple systemic side effects, can be avoided.
